# Shift Work Disorder in a Random Population Sample – Prevalence and Comorbidities

**DOI:** 10.1371/journal.pone.0055306

**Published:** 2013-01-25

**Authors:** Lee Di Milia, Siri Waage, Ståle Pallesen, Bjørn Bjorvatn

**Affiliations:** 1 School of Management and the Institute for Health and Social Science Research, Central Queensland University, Rockhampton, Queensland, Australia; 2 Norwegian Competence Center for Sleep Disorders, Haukeland University Hospital, Bergen, Norway; 3 Department of Public Health and Primary Health Care, University of Bergen, Bergen, Norway; 4 Department of Psychosocial Science, University of Bergen, Bergen, Norway; University of Alabama at Birmingham, United States of America

## Abstract

Few studies have investigated the presence of shift work disorder (SWD) in the general community. We addressed many of the limitations in this literature and present new findings. SWD has been treated as an ‘all or none’ construct but we propose the need to consider the ‘severity’ of the disorder. Using random digit dialling, we randomly recruited 1163 participants. Participants completed an extensive battery of scales and questions concerning work, health and individual differences. Three questions based on the criteria from the International Classification for Sleep Disorders were used to categorise participants with SWD (n = 176). In addition, we asked participants whether SWD interfered with aspects of their life and high ratings were used to define severe shift work disorder (SSWD). The prevalence of SWD was 32.1% among night workers and 10.1% in day workers (p<.001). SSWD was present in 9.1% of night workers and 1.3% of day workers (p<.001). Adjusted logistic regression analyses found significant associations between SWD and night work (OR  = 3.35, CI 2.19-5.12), weekly work hours (OR  = 1.02, CI 1.00–1.04), short sleep (≤6 h; OR  = 2.93, CI 1.94–4.41), languidity (OR  = 1.11, CI 1.06–1.16) and resilience (OR  = 0.56, CI 0.43–0.81). Night work, short sleep, languidity, and hypertension were significantly associated with SSWD. Overall, participants with SSWD slept 0.80 h less than other participants (p<.001). Night work, short sleep and languidity were associated with both SWD and SSWD. Day workers with SWD symptoms reported significantly shorter sleep duration, higher levels of languidity and worked longer working hours compared to day workers without SWD.

## Introduction

Approximately 16% of Australian employees are employed on some form of work schedule that includes working during the night [1]. A consistent finding is that working night shift is associated with shorter sleep duration and increased sleepiness [2], [3]. Compared to day workers, night workers have been reported to sleep from 15 minutes [4] to two hours less per day [5]. Objective indicators of sleepiness show that sleepiness increases across the night shift [6] and falling asleep during night work is not uncommon [2]. In addition, there is an increasing recognition that night work may play a role in the development of metabolic syndrome (MetS) [7]. MetS may be considered as a cluster of risk factors (e.g. obesity, hypertension, hypercholesterolemia) that contribute to poor health [8].

Many studies have focussed on the effect of sleepiness on reaction time [9] and driving performance [10], still very few studies have specifically investigated shift work disorder (SWD); a condition characterised by excessive sleepiness and insomnia due to unfavourable work hours. The 2^nd^ edition of the *International Classification of Sleep Disorders* sets out the following criteria to diagnose SWD: 1) complaints of insomnia or excessive sleepiness temporarily associated with a recurring work schedule that overlaps the usual time for sleep; 2) symptoms must be associated with the work schedule for at least one month; 3) evidence that the circadian and sleep-time misalignment were present for ≥ 7 days using sleep log or actigraphic recording; and, 4) the sleep disturbance cannot be explained by another sleep, medical, neurological or mental disorder, or the result of medication or substance abuse [11].

The prevalence of SWD shows a good deal of variation reflecting the criteria employed to define the construct and arguably, the unique characteristics of the samples. Drake et al. [12] used the Epworth Sleepiness Scale (EES) [13] to assess sleepiness and criteria based on the Diagnostic and Statistical Manual of Mental Disorders [14] to measure insomnia. The prevalence of insomnia and/or sleepiness was 32% in permanent night workers, 26% in rotating shift workers and 18% in day workers. The prevalence of ‘true SWD’ in night and shift workers was calculated at 10%; and reflected the discrepancy between insomnia/sleepiness in these groups compared to day workers. One major limitation of this study is that respondents were not directly asked if the insomnia or sleepiness were related to their work schedule. Furthermore, the authors did not consider the possibility of suffering from SWD when working day shifts (e.g., with very early start times).

Rajaratnam et al. [15] estimated that SWD was present in 14.5% of police officers that worked night shift. This estimate was derived by defining SWD in terms of excessive sleepiness and insomnia. However, this estimate increased markedly to 54% when the ICSD-2 criteria (excessive sleepiness *or* insomnia) were applied. Sleepiness was here assessed in terms of the chances of dozing or falling asleep during night work or commuting following night work compared with days off. Insomnia was classified using the Athens Insomnia Scale [16]. SWD was associated with an increased risk of falling asleep while driving and during meetings, absenteeism and anger in dealing with suspects.

Waage et al. [17] developed three questions (see Methods) that map onto the ICSD-2 criteria and reported that SWD was present in 23% of shift-working oil-rig workers. These workers reported poor sleep quality, greater health complaints and less ability to cope compared to those without SWD. However, many reported that SWD did not have a major impact on their lives. In a large study of nurses, Flo et al. [18] employed three different approaches to identifying SWD. One method used the questions developed by Waage et al. The second operationalization combined these questions with elevated scores on the Bergen Insomnia Scale [19] and/or the ESS [13], whereas the third at the same time excluded respondents reporting symptoms of specific other sleep disorders assessed by the Global Sleep Assessment Questionnaire [20]. The three approaches produced estimates of SWD between 32% and 37%.

There is some evidence linking SWD with a number of comorbidities. Permanent night workers and rotating shift workers with SWD were reported to have more ulcers, sleep related accidents, absenteeism, neuroticism, depression and shorter sleep durations compared to those without SWD [12]. Comorbidities have also been found in other sleep disorders. Individuals with obstructive sleep apnea were reported to have higher adjusted odds ratios for diabetes, cardiovascular disease, gastrointestinal tract disorders, lower health status and burnout [15]. Some of these outcomes are also associated with MetS. In a review of the literature Wang et al. [7] concluded that the data support a ‘moderate’ link between rotating shift work and MetS. The role of sleep loss in the development of SWD and MetS suggests a common pathway.

Against this backdrop we conducted an epidemiological study to address some of the limitations in the SWD literature. First, we ensured the symptoms of SWD were a function of the work schedule. Second, the ability to generalise the presence of SWD in the community is limited because the previous studies have focussed on narrow occupational groups. In contrast, we based this study on a general population with a wide range of occupations. Third, SWD has been considered as an ‘all or none’ construct but we propose its impact would vary by its severity. Thus, we extend the literature by examining the relationship with a number of variables and severe SWD (SSWD). Fourth, it may be the case that individual differences play a role in the development of SWD but fewer studies have examined these relationships [18]. Finally, we explore the association between MetS and SWD.

## Methods

### Ethics Statement

Participants were contacted by telephone and were informed that the aim of the study was to examine the relationship between work and well-being. In addition, participants were informed that participation was voluntary, confidential and they were free to withdraw consent at any time during the interview. Participants were not remunerated for their time. Contacting participants by telephone made it problematic to obtain written consent. However, participants were asked to indicate their verbal consent prior to the interview and this decision was recorded in the database. The study and the requirement to obtain verbal agreement were approved by the Human Research Ethics Committee at Central Queensland University.

### Procedure

We conducted a telephone survey using random digit dialling in three Australian regional cities. Interviewers asked to speak to someone over the age of 18 in some form of paid employment. The survey was conducted by trained interviewers not associated with the study.

### Measures

Participants completed a comprehensive battery of questions and scales. We collected demographic data and work schedule details. We asked about daily and weekly work hours and, whether they worked primarily during the day, afternoon or night, as well as combinations of these shifts in order to classify workers. Participants that worked during the day, afternoon or a mix of day and afternoon shifts were classified as day workers (n = 898) and those working rotating shifts including night work were classified as night workers (n = 265). Self-reported height and weight were used to calculate body mass index (BMI).

We categorised SWD on the basis of answering ‘yes’ to each of the following questions [17]: 1) Do you experience difficulties in sleeping or excessive sleepiness? 2) Do you regard your sleep or sleepiness problem to be related to your work schedule? 3) Has this sleep or sleepiness problem related to your work schedule persisted for at least one month? A key feature of these questions is the focus on the work schedule and thus is applicable to both day and night workers. Participants who endorsed these three items were further asked to indicate to which degree this sleep or sleepiness problem negatively affected their social, family or work relationships (1 = not at all; 5 = very much). A rating of ‘very much’ was used to create the SSWD group.

Participants completed a number of scales that through previous research are reported to be reliable and valid:

#### Revised Circadian Type Inventory (rCTI)

The rCTI contains two scales that measure different aspects of the circadian rhythm [21]. The languid-vigour (LV) scale provides information concerning amplitude. A sample item is, ‘*if you have to get up very early one morning do you tend to feel tired all day?’* High scores on languid suggest difficulties with night work. The stability of the rhythm is measured using the flexible-rigid (FR) scale and high scores on flexibility suggest better adjustment to night work. A sample item is, *‘do you enjoy working at unusual times of day or night?’* Cronbach alpha (α) for the LV scale was .67 and .72 for the FR scale.

#### Morning Affect (MA)

The MA scale is a pure measure of morningness containing four items that assess morning behaviour [22]. A sample item is, *‘how alert do you feel during the first half hour after having woken in the morning?’* High scores suggest greater morningness (α = .82).

#### Brief Resilience Scale (BRS)

The BRS is a six item scale [23]. A sample item is, ‘*I tend to bounce back quickly after hard times.’* Higher scores suggest better resilience (α = .79).

#### Brief Resilience Coping Scale (BRCS)

The BRCS is a four item scale [24]. A sample item is, ‘*regardless of what happens to me, I believe I can control my reaction to it.’* Higher scores suggest better coping (α = .61).

We also asked a series of single item questions concerning: typical sleep obtained per day (excluding time in bed) and consistent with the literature we categorised short sleep duration as ≤6 h [25]; job satisfaction (1 = very dissatisfied; 5 = very satisfied); whether usual work tasks were physically demanding (1 = very low; 7 = high); general health (1 = excellent; 5 = poor); whether they had been diagnosed by a doctor with the following conditions (yes/no): depression, hypertension, hypercholesterolemia, irritable bowel syndrome, asthma/chronic bronchitis and cancer; educational status: some studies have suggested that people working nights and people with MetS have lower educational qualifications [8], [26]. We coded our sample to reflect whether they had attained university qualifications or not.

### Data Analyses

SPSS (Ver. 20) was used to conduct all analyses. Chi-square tests were used to compare categorical data. We conducted a multivariate analysis of variance (ANOVA) using the factors SWD (yes, no) and shift type (day, night). The four resulting groups were compared on a number of variables. Significant mean differences resulting from the interaction effects were examined post-hoc using one-way ANOVA and the conservative Scheffe test.

Next, we computed the odds ratio (OR) and the 95% confidence interval using two sets of logistic regression analyses to separately determine the associations with SWD and SSWD. For each analysis we first used a bivariate model to assess the association between SWD and a number of variables. The variables that were significantly associated with SWD were then included using the ‘enter’ method into a second model to produce a fully adjusted model. This two-step process was also used to determine the associations with SSWD. We investigated any possible multicollinearity between the predictor variables and found no indication of multicollinearity.

## Results

We contacted 2323 individuals and 1194 interviews were completed. Of these, the employment status of 31 participants was missing and these records were consequently deleted. After deletion we analysed the data from 1163 interviews (50% response rate).

The sample consisted of 540 (46%) males and 623 females. The mean age was 45.3 years (SD = 11.2) and gender differences on age were not significant. A comparison of the day and night work groups with, and without SWD can be found in Table 1. Pillai's trace suggested a significant main effect for SWD (p<.001) and shift type (p<.001), as well as the interaction between these factors (p<.013). The absence of interaction effects suggested that the groups were similar for age, general health, BMI, morningness, job satisfaction and physical job demand. However, day workers with SWD reported significantly less sleep per day, were more languid and worked longer daily and weekly hours compared to day workers without SWD. Night workers with SWD reported significantly more languidity than night workers without SWD but were similar for sleep duration and working hours.

**Table 1:** Descriptive statistics describing day and night workers with and without shift work disorder (SWD).

	Shift Work Disorder (SWD)		No Shift Work Disorder (NSWD)		
Continuous variables*	Day work	Night work	Day work	Night work	p**
Mean ± SD	(n = 81; 8.0%)	(n = 689; 68.1%)	(n = 77; 7.6%)	(n = 165; 16.3%)	
Age	44.52 (10.95)	45.10 (11.87)	45.23 (10.94)	45.95 (11.02)	.947
General health	3.00 (0.91)	3.12 (0.90)	2.71 (0.94)	2.75 (0.93)	.638
BMI	29.45 (5.95)	28.82 (5.47)	27.63 (5.54)	28.60 (5.81)	.115
Sleep duration (hours) 1	6.49 (0.87)	6.61 (1.19)	7.02 (1.05)	6.75 (1.07)	.040
Languid^2^	16.99 (5.45)	19.32 (5.16)	14.14 (5.38)	14.22 (4.77)	.018
Flexible	12.61 (4.89)	15.87 (5.49)	13.23 (5.15)	17.63 (4.84)	.216
Morningness	10.69 (2.94)	10.71 (3.07)	12.51 (2.61)	12.38 (2.39)	.751
Resilience	3.16 (0.79)	3.28 (0.69)	3.56 (0.68)	3.71 (0.57)	.809
Job satisfaction	3.63 (1.03)	3.58 (1.13)	4.15 (0.90)	4.10 (0.98)	.992
Physical job demands	3.90 (1.83)	3.97 (1.95)	3.56 (1.98)	4.24 (1.99)	.090
Work hours/week^3^	44.01 (12.71)	42.97 (13.41)	37.60 (13.82)	42.74 (16.21)	.016
Work hours/day^4^	8.74 (2.05)	9.48 (2.82)	7.85 (2.13)	9.85 (2.71)	.001

Categorical variables*	(n = 91; 7.8%)	(n = 807; 69.4%)	(n = 85; 7.3%)	(n = 180; 15.5%)	
Males	42 (46.2%)	61 (71.8%)	317 (39.3%)	120 (66.7%)	.001
Females	49 (53.8%)	24 (28.2%)	490 (60.7%)	60 (33.3%)	
Work full-time	78 (85.7%)	68 (80.0%)	531 (65.8%)	141 (78.3%)	.001
Work part-time	42 (14.3%)	17 (20.0%)	276 (34.2%)	39 (21.7%)	
Hypertension – no	66 (72.5%)	62 (72.9%)	663 (2.2%)	141 (78.3%)	.001
Hypertension – yes	25 (72.5%)	23 (27.1%)	144 (17.8%)	39 (21.7%)	

* Sample size: continuous variables  = 1012, categorical variables  = 1163.

** interaction effects (SWD x Shift Type).

1 SWD day shift significantly < sleep than NSWD day shift; NSWD day shift > longer sleep than other means; SWD night shift significantly < than NSWD day shift; NSWD night shift significantly < than NSWD day shift.

2 SWD day shift significantly > higher than NSWD day shift and NSWD night shift; SWD night shift significantly > than NSWD day shift; SWD night shift significantly > higher than NSWD night shift.

3 SWD day shift significantly > longer hours than NSWD day shift; NSWD day shift significantly < hours than SWD night shift and NSWD night shift.

4 SWD day shift significantly > longer hours than NSWD day shift, and significantly < fewer hours than NSWD night shift; NSWD day shift significantly < hours than SWD night shift and NSWD night shift.

Overall 176 participants met the criteria for SWD (15%). The prevalence was greater in the night work group (32.1%) compared with day workers (10.1%) group (χ^2^ = 76.71, df  = 1, p<.001). Males (19.1%) were also more likely to report SWD than females (11.7%; χ^2^ = 12.19, df  = 1, p<.001). Approximately 20% of the SWD sample reported that the condition impacted ‘very much’ on their social, family and work relationships. We conducted a univariate general linear model and the result suggested that sleep duration significantly decreased *F*(4, 174)  = 3.10, p<.017, η^2^ = .07 as the severity of SWD increased (see Table 2).

**Table 2:** Impact of shift work disorder on day time function and sleep duration (n = 176*).

Impact	N (Percent)	Sleep duration (M ± SD)
Not at all	12 (6.8)	7.08±0.79
A little	35 (19.9)	6.69±1.30
Somewhat	42 (23.9)	6.76±1.08
Moderately	50 (29.0)	6.42±0.88
Very much	36 (20.5)	6.14±0.87

* the sample contained day and night time workers.

Applying our criteria to define SSWD resulted in a prevalence of 3.1% (n = 36). The prevalence was 9.1% among night workers and 1.3% among day workers (χ^2^  = 40.66, df  = 1, p<.001). Sleep duration was significantly less among those with SSWD (6.14±0.87) compared with all other participants (6.94±1.06).

The bivariate OR suggested significant associations between SWD and a number of variables (see table 3): night work, weekly work hours, male, languidity, morningness, coping, resilience, body mass index, depression, hypertension, asthma/bronchitis and short sleep. Many of these variables (except coping, body mass index and depression) and the addition of hypercholesterolemia and irritable bowel syndrome were significantly associated with SSWD. The associations between the predictor variables, SWD and SSWD resulting from the fully adjusted logistic models can be found in Table 4. Night work (p<.001), weekly work hours (p<.024), languidity (p<.001) and short sleep (p<.001) were positively associated with SWD, while resilience (p<.001) was negatively associated with SWD. Night work (p<.002), languidity (p<.001), short sleep (p<.001) and hypertension (p<.009) were significantly associated with SSWD. The predictor variables in both models were not highly correlated. Using linear regression the statistics for the variance inflation factor ranged between 1.02 and 1.80, while tolerance ranged between 0.56 and 0.98.

**Table 3:** Unadjusted odds ratio (OR) and confidence intervals (CI) for variables associated with shift work disorder and severe shift work disorder.

	Shift work disorder			Severe shift work disorder		
Variable	OR	CI (95%)	p	OR	CI (95%)	p
Age	1.00	0.98 – 1.01	.621	1.03	1.00 – 1.06	.054
Gender: female	1.00			1.00		
Gender: male	1.78	1.28 – 2.46	.001	2.37	1.17 – 4.78	.008
Night work: No	1.00			1.00		
Night work: Yes	4.19	2.99 – 5.87	.001	7.35	3.62 – 14.92	.001
Weekly work hours	1.03	1.02 – 1.04	.001	1.03	1.00 – 1.05	.001
Physical work load	1.11	0.76 – 1.62	.586	1.36	0.65 – 2.05	.423
Languid	1.14	1.11 – 1.17	.001	1.19	1.12 – 1.26	.001
Flexible	1.01	0.98 – 1.04	.419	1.00	0.94 – 1.07	.928
Morningness	0.81	0.77 – 0.86	.001	0.84	0.75 – 0.93	.001
Coping	0.94	0.89 – 0.99	.017	0.94	0.85 – 1.05	.269
Resilience	0.48	0.38 – 0.60	.001	0.48	0.31 – 0.73	.001
Body mass index	1.04	1.01 – 1.07	.001	1.04	0.99 – 1.10	.133
Depression: No	1.00			1.00		
Depression: Yes	1.63	1.12 – 1.37	.011	1.47	0.68 – 3.18	.324
Hypertension: No	1.00			1.00		
Hypertension: Yes	1.65	1.14 – 2.38	.001	3.01	1.53 – 5.94	.001
Asthma/chronic bronchitis: No	1.00			1.00		
Asthma/chronic bronchitis: Yes	1.73	1.14 – 2.63	.011	2.26	1.04 – 4.90	.039
Hypercholesterolemia: No	1.00			1.00		
Hypercholesterolemia: Yes	1.35	0.91 – 2.00	.135	3.12	1.57 – 6.20	.001
Irritable Bowel: No	1.00			1.00		
Irritable Bowel: Yes	1.79	0.94 – 3.42	.075	3.47	1.30 – 9.31	.013
Education: University level	1.00			1.00		
Education: < University	1.11	0.79 – 1.55	.546	0.83	0.42 – 1.65	.602
Sleep: > 6h	1.00			1.00		
Sleep: ≤ 6h	2.80	2.02 – 3.88	.001	8.80	3.12 – 20.29	.001
Cancer: No	1.00			1.00		
Cancer: Yes	0.99	0.60 – 1.62	.261	0.72	0.10 – 5.38	.749

**Table 4:** Adjusted odds ratio for predictor variables associated with shift work disorder and severe shift work disorder.

	Shift work disorder (n = 176)			Severe shift work disorder (n = 36)		
	Odds ratio	CI (95%)	p	Odds ratio	CI (95%)	p
Gender: female	1.00			1.00		
Gender: male	1.41	0.88 – 2.26	.155	1.86	0.74 – 4.69	.187
Night work: No	1.00			1.00		
Night work: Yes	3.35	2.19 – 5.12	.001	3.83	1.66 – 8.83	.002
Weekly work hours	1.02	1.00 – 1.04	.024	1.02	0.99 – 1.05	.191
Languid	1.11	1.06 – 1.16	.001	1.28	1.15 – 1.42	.001
Morningness	0.93	0.85 – 1.01	.097	1.12	0.94 – 1.32	.200
Resilience	0.59	0.43 – 0.81	.001	0.91	0.51 – 1.63	.749
Hypertension: No	1.00			1.00		
Hypertension: Yes	1.56	0.97 – 2.49	.066	3.30	1.35 – 8.05	.009
Asthma/chronic bronchitis: No	1.00			1.00		
Asthma/chronic bronchitis: Yes	1.62	0.96 – 2.70	.068	1.61	0.62 – 4.17	.325
Sleep > 6h	1.00					
Sleep ≤ 6h	2.93	1.94– 4.41	.001	9.11	3.56 – 23.31	.001
Body mass index	1.03	0.99 – 1.06	.121	Not included*		
Coping	1.00	0.94 – 1.07	.968	Not included		
Depression: No	1.00					
Depression: Yes	1.21	0.72 – 2.00	.473	Not included		
Hypercholesterolemia: No	Not included			1.00		
Hypercholesterolemia: Yes				2.00	0.84 – 4.73	.116
Irritable Bowel: No	Not included			1.00		
Irritable Bowel: Yes				2.81	0.83 – 9.57	.098

* Not included means these predictor variables were not significantly associated with shift work disorder or severe shift work disorder at the bivariate level and therefore, not included in the adjusted model.

## Discussion

There are relatively few studies of SWD and many have some limitations that we attempted to remedy. In particular, we ensured that the symptoms of SWD were linked to the work schedule, we included participants drawn from the population to generalise the results and we extended the literature to consider the severity of SWD. Furthermore, we assessed the associations between individual differences and SWD, and the link between SWD and MetS.

The prevalence of SWD among Australian night workers was 32% and this compares favourably with figures of 26% obtained from rotating shift workers and 32% from permanent night workers [12]. The similarity between these results may be the result of using random population samples because the studies employed different criteria to define SWD.

Compared to studies that employed the same methods to define SWD [17], [18] our estimate fell between the prevalence reported in these occupation specific studies. The lower estimate based on oil-rig workers may reflect the fact that these workers undergo regular health screens and are only allowed to work when assessed to be physically fit. Furthermore, these workers live in an environment that is free from domestic demands which may create more recovery time. Studies have also shown that oil-rig workers obtain good biological adaptation to night work [6] in contrast to other night workers [27]. These factors may also explain why only a small number of oil-rig workers reported that SWD was problematic to their lifestyles [17]. The higher SWD estimate derived from a primarily female nursing sample may be because females still carry the bulk of the domestic burden. In addition, nursing schedules often involve quick returns from afternoon to morning shifts that limit work recovery time [18]. One limitation of the ICSD-2 criteria that is used to define SWD is that it does not recognise the severity of the condition. Applying our criteria for severe SWD resulted in a prevalence of approximately 9.1% of night- and 1.3% of day workers in the sample.

Working nights, short sleep and languidity were significantly associated with both SWD and SSWD. The associations with night shift and short sleep may not be too surprising given that a number of studies have linked night work and sleep loss with SWD [4], [5]. However, our results suggest the contribution and magnitude of these two variables differs according to the severity of the disorder and this may have implications for treatment. The OR suggested that night work had the strongest association with SWD while short sleep was a little weaker. However, when we considered the associations with SSWD, the OR suggested that short sleep was the most important variable (OR  = 9.11, 95%CI 3.56–23.31, p<.001) followed by night work (OR  = 3.83, 95%CI 1.66–8.83, p<.001). The strength of the association with short sleep should be interpreted with some caution because of the large confidence interval surrounding the OR. This result reflects the small number of people classified with SSWD.

A number of studies [12], [17], [18] have reported a dose-response relationship between night work and SWD and this suggests that reducing exposure to night shift may ameliorate the impact of this condition. Weekly work hours were also significantly associated with SWD. Long work hours are inversely related to sleep duration [28] and therefore, may contribute to the development of SWD by decreasing the amount of time available for sleep. In support of this argument we found that those with SSWD slept significantly less. However, the association between weekly work hours and SSWD was not significant in the adjusted model. This may be due to the much smaller number of people reporting SSWD.

Increasing sleep length would seem to be one solution to mitigate SSWD but this suggestion may be too simplistic given sleep duration is influenced by a number of factors. Our study was not designed to investigate the reasons for short sleep duration and future studies should aim to shed further light on the factors that contribute to short sleep. It is clear that SWD sufferers report more chronic sleepiness [12], poor quality sleep and greater sleep disturbances [17].

An interesting finding from our results and others [12] is that day workers also report symptoms associated with SWD. On one hand, this finding may suggest some changes to the criteria to diagnose SWD. However, it may also be argued that day work that commences too early in the morning is shift work. More recent conceptualisations of working arrangements suggest that shift work (or irregular work arrangements) may be applied to work that occurs outside of the hours between 07:00 and 18:00 [29]. Day workers that rise too early report shorter sleep durations and greater sleepiness [30]. Our results supported the conclusion that day workers with SWD symptoms obtained significantly less sleep than day workers without SWD.

Languid types are considered to cope less well with night work [21] and our results were consistent with this notion. Languid types were significantly linked with SWD and SSWD and our result adds to the growing number of studies finding a negative relationship between night work and languidity [18], [31]. We suggest that this measure could be used as a counselling tool for night workers. Resilience was found to be negatively linked with SWD and this suggests that resilient types are able to deal with moderate levels of SWD but not SSWD. This raises the possibility that increasing resilience may assist in decreasing SWD. A wider discussion of interventions can be found elsewhere [32].

SWD has been linked with a number of comorbidities [15] and we proposed a possible link with MetS. The crude OR identified significant associations between SWD, BMI and hypertension but this relationship was not supported in the adjusted model. Hypertension was however, significantly associated with SSWD. One possible explanatory variable is sleep duration. Gottlieb et al. [33] reported for example an inverse relationship between sleep loss and hypertension. Our results are consistent with other studies [12] in finding less sleep among people with SWD.

The results need to be considered in the context of the strengths and weaknesses of this study. We conducted an epidemiological random population based study and employed several reliable and valid scales. Our approach to assessing SWD meets the core criteria for its diagnosis [11]. It was not possible to conduct clinical interviews or employ actigraphy given our large sample but our questions concerning SWD are arguably the questions a clinician typically would use to assist in making a diagnosis. In line with this, it has been suggested that SWD may be diagnosed using anamnestic data alone [34]. Our response rate (50%) was within the range of 60±20% suggested by Baruch [35] as not requiring further investigation. Non-responders often have an impaired health status [36] and this suggests that our estimates may actually be conservative. Nonetheless, the absence of a full response rate suggests the possibility of bias. Another limitation is that the cross-sectional design of the study does not allow causal inferences to be made. We recommend that future studies aim to better understand the pathways that lead to SWD. The use of single items to measure some constructs including medical conditions may be a limitation. However, there is evidence that single items are reliable indicators [37] and concerning the medical conditions, we asked whether these were diagnosed by a doctor. This gives greater confidence that the participants were in fact diagnosed with these conditions rather than asking for an individual's self-report which is still common in the literature [38]. As all data were based on self-report, the common method bias may have influenced the findings [39]. An additional limitation of the study is that we did not consider the contribution of shift work exposure (i.e. years in night work) or the actual shift start times. Exposure to shift work is associated with an increased prevalence of metabolic disorders [40] and sleep impairments. A ten year follow up study of French workers found that exposure to shift work was linked with disrupted sleep, difficulty returning to sleep and premature awakening [41]. Some of these impairments were diminished following retirement. The actual shift start times may explain why some day workers reported SWD symptoms. A number of studies have demonstrated that sleepiness increases and sleep duration deceases as morning wake time is advanced [29], [42]. Our results suggested that day workers with SWD obtained significantly less sleep but we do not know whether this is because they woke earlier or because of some other factor.

In conclusion, one-third of night workers had symptoms consistent with SWD and 9% of night workers reported severe SWD. Night work, short sleep and languidity were significantly associated with SWD and severe SWD. Reducing night work exposure appears to be the best intervention strategy. We found no link between MetS and SWD but a significant association was found between hypertension and severe SWD.
